# Prediction of hospitalization and waiting time within 24 hours of emergency department patients with unstructured text data

**DOI:** 10.1007/s10729-023-09660-5

**Published:** 2023-11-03

**Authors:** Hyeram Seo, Imjin Ahn, Hansle Gwon, Hee Jun Kang, Yunha Kim, Ha Na Cho, Heejung Choi, Minkyoung Kim, Jiye Han, Gaeun Kee, Seohyun Park, Dong-Woo Seo, Tae Joon Jun, Young-Hak Kim

**Affiliations:** 1 Department of Medical Science, Asan Medical Institute of Convergence Science and Technology, Asan Medical Center, University of Ulsan College of Medicine, 88, Olympicro 43gil, 05505 Seoul, Songpagu Korea; 2 Division of Cardiology, Department of Information Medicine, Asan Medical Center, University of Ulsan College of Medicine, 88, Olympicro 43gil, 05505 Seoul, Songpagu Korea; 3 Department of Emergency Medicine, Asan Medical Center, University of Ulsan College of Medicine, 88, Olympicro 43gil, 05505 Songpagu Seoul, Korea; 4 Big Data Research Center, Asan Institute for Life Sciences, Asan Medical Center, 88, Olympicro 43gil, 05505 Songpagu Seoul, Korea

**Keywords:** Hospital admission prediction, Electronic medical record, Emergency department, Machine learning, Explainable artificial intelligence, Natural language processing

## Abstract

Overcrowding of emergency departments is a global concern, leading to numerous negative consequences. This study aimed to develop a useful and inexpensive tool derived from electronic medical records that supports clinical decision-making and can be easily utilized by emergency department physicians. We presented machine learning models that predicted the likelihood of hospitalizations within 24 hours and estimated waiting times. Moreover, we revealed the enhanced performance of these machine learning models compared to existing models by incorporating unstructured text data. Among several evaluated models, the extreme gradient boosting model that incorporated text data yielded the best performance. This model achieved an area under the receiver operating characteristic curve score of 0.922 and an area under the precision-recall curve score of 0.687. The mean absolute error revealed a difference of approximately 3 hours. Using this model, we classified the probability of patients not being admitted within 24 hours as Low, Medium, or High and identified important variables influencing this classification through explainable artificial intelligence. The model results are readily displayed on an electronic dashboard to support the decision-making of emergency department physicians and alleviate overcrowding, thereby resulting in socioeconomic benefits for medical facilities.

**Supplementary Information:**

The online version contains supplementary material available at 10.1007/s10729-023-09660-5.

## Highlights

- We created ML models that predict hospitalization and waiting time through information from patients who visited ED and hospitalization decision forms
- Unstructured text data improves hospitalization prediction AUROC by 6%
- Explainable artificial intelligence showed that text variables have a high influence on prediction results
- We provide a useful inexpensive tool for ED physicians to make quick decisions

## Introduction

In addition to the emergency department (ED) serving as a key central access point for cases of critical emergencies, it functions as a primary health care system for patients who present with non-critical, concerning symptoms without an alternative option for outpatient care [1]. The overcrowding of EDs occurs as a result of the lack of hospital beds, distorted nurse-to-patient ratios, diagnostic errors, and ambulance diversions to the EDs, as well as delays in diagnostic and procedural practices [2–4]. ED overcrowding has negative consequences, including increased mortality rates, longer ED lengths of stay, treatment errors, lower rates of patient satisfaction, and challenges related to ambulance availability [5–7]. Moreover, as the medical industry is service-oriented, patient dissatisfaction is correlated with a decline in ED consultations and an unfavorable perception of hospitals. Consequently, the operation of EDs may be additionally adversely affected, thus impacting the hospital’s financial management [8].

Under the governance of the Ministry of Health and Welfare of South Korea, there are numerous operational medical institutions and health systems. Furthermore, the emergency medical system in Korea functions through the collaboration of emergency medical technicians, centers, institutions, and rooms. The emergency medical response system comprises accident scene management, transportation, stages, and communication systems. Among these stages, the hospital care stage occurs at the level of the emergency medical center [9]. The healthcare system in South Korea aims to qualitatively improve and expand emergency medical services through the “Comprehensive Plan for Emergency Medical System Improvement” policy initiative. The government has provided policy support to achieve this goal, and the decisions arising from this initiative have considerably influenced EDs nationally. In South Korea, the “24-hour Emergency Department Restriction Act” was enacted in December 2017 for upper-level general hospitals to address the need for the quick examination and diagnosis of critically ill emergency department patients, as well as the resolution of overcrowding in the ED. According to this law, the percentage of patients with an ED length of stay exceeding 24 hours should be maintained within 5% annually. Additionally, in 2004, South Korea introduced the National Emergency Department Information System (NEDIS) to computerize and manage the medical records of patients presenting at emergency medical facilities. This system enables real-time tracking and sharing of the emergency conditions and treatment details of patients. In 2017, by utilizing the medical records collected by NEDIS, treatment delays for critically ill patients, overcrowding in emergency rooms, and lack of diagnostic reliability were nationally addressed by assessing this data using the Korea Triage and Acuity Scale (KTAS). KTAS is a comprehensive patient classification system that spans from the pre-hospitalization to in-hospital stages, determining patient priorities and urgency levels. These efforts have led to the emergence of specialized hospitals that evaluate various medical facilities, healthcare systems, and medical data. These hospitals serve as the foundation for research and technological development to enhance South Korea’s emergency medical system [10].

In this study, our motivations included contributing to the emergency medical system and alleviating overcrowding in the ED, thus improving the ED environment. To act on these motivations, we implemented the following steps: First, we developed models that could hypothetically predict the likelihood of whether ED patients would require hospital admission and estimate the waiting time [11]. This was theorized to enable support for the decision-making process of ED physicians, allowing for the minimization of the proportion of patients with an ED length of stay exceeding 24 hours. Second, we compared the performance of a training model with natural language processing (NLP) generated variables, from unstructured text data to models without it. The free handwritten text notes of ED doctors and nurses are a mixture of Korean, English, numerals, and symbols, conveying a substantial amount of information [12]. Consequently, the rapid reproducibility and easy maintenance of hospitalization prediction models emphasized the importance of text processing and comparing the performance of models. Finally, we utilized explainable artificial intelligence (XAI) to distinguish ineligibility factors, explaining why the patient could not be admitted. Thus, this allowed for identifying departments with waiting time delays and minimizing these waiting times by improving patient flow.

**Fig. 1:**
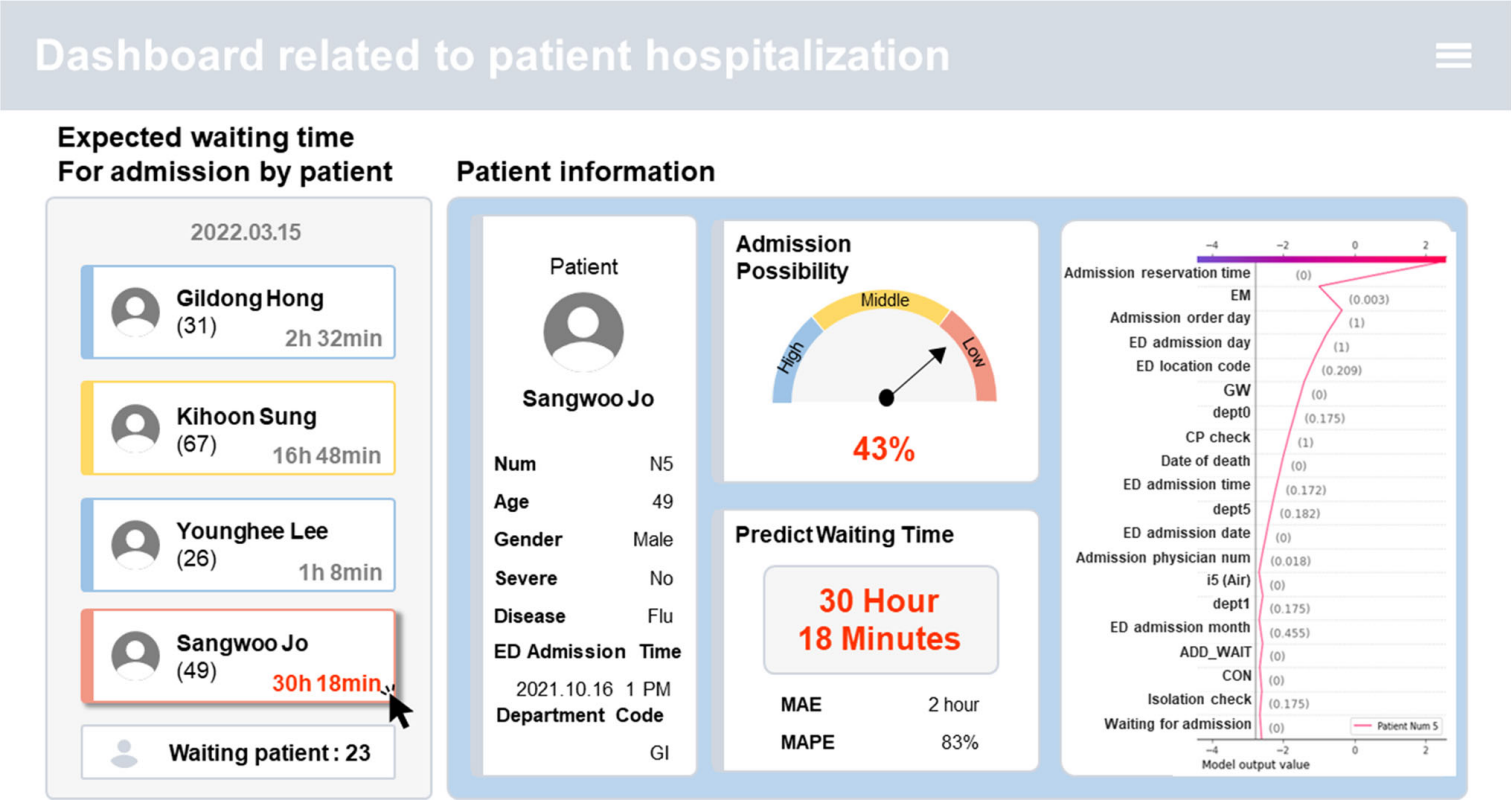
A synthesis of the findings of this study: A virtual visualization dashboard depicting the prediction of the likelihood of hospitalization and estimated waiting times for patients admitted to the ED. A patient’s likelihood of hospitalization is indicated by blue (High), yellow (Medium), and red (Low) in order of probability. Displayed in the table on the right, in order of importance from the top, are the causes of patients not hospitalized within 24 hours of presenting to the ED (Please note that the names of the patients provided are hypothetical, as only de-identified data were extracted)

Therefore, to recapitulate, we aimed to develop a practical and cost-effective tool to assist ED physicians in making clinical decisions using electronic medical records (EMR). Recently, Johns Hopkins Hospital introduced a command center that utilizes an electronic dashboard to facilitate systematic communication with hospital staff [13]. Figure 1 represents a visualization of a virtual dashboard to potentially be applied in a hospital, integrating the results of this study. This visualization dashboard is designed to be practical for ED physicians. Displaying real-time information to medical staff and informing patients about the estimated waiting time on the screen reduces the number of ED consultations and lessens waiting time in the operating room. Furthermore, forbearance is improved, and anxiety is reduced in patients. Providing patients with delayed information diminishes hospital revenue losses by preventing them from leaving the hospital abruptly [14, 15].

In the Related Works section below, an overview of the existing literature addressing overcrowding in EDs has been performed. The Methods section covers the datasets used, pre-processing steps, training and evaluation methods, explanations of artificial intelligence (AI) model algorithms, and XAI techniques. In the Results section, the findings and performance metrics of this study have been presented. The Discussion section provides an interpretation of the results of this study, highlights its limitations, and suggests scope for improvement. Finally, the Conclusion section provides an overall summary of this study, in addition to a detailed explanation of its clinical implications.

## Related works

Patients often perceive the waiting time for admission to be longer than the actual elapsed time, and reducing this perceived waiting time has been shown to increase patient satisfaction. Various studies have suggested several methods to reduce the perceived waiting time of patients. These potential strategies include improving interpersonal interactions, providing patients with information and guidance on appropriate waiting times, and considering changes in staffing levels [16–18]. Physicians need to counsel patients regarding lengthy waiting times until admission or when hospital admission is not possible. Large data and predictive analytics from EDs improve the efficiency of emergency medical services and enhance the treatment of patients. This results in personalizing patient care, improved efficiency, and limited wasteful spending by providing practical guidance without investing in extra resources [19]. To do this, we require efficient and cost-effective decision-making tools to help ED physicians [20].

One technique to alleviate overcrowding is to admit the appropriate ED patients to the hospital promptly. Numerous studies have been conducted on the admission prediction for mitigating ED overcrowding. A high-performance logistic regression (LR) admission prediction model incorporating demographics, management, and clinical data can be routinely obtained in a hospital, informing patients about the likelihood of their admission [21]. One model uses patient information commonly acquired during ambulance transportation [22]. Based on the baseline characteristics provided on presenting at an ED, yet another predictive model divides a patient’s electronic health record (EHR) dataset regarding statistics on prior medical use, past medical histories, insurance companies, and employment agencies into three categories (triage, history, full) [23]. The optimal waiting time for patients with low acuity grades using historical patient data, such as a machine learning (ML) tree-based predictive model, has been predicted [8]. Moreover, a model exists for predicting the expected waiting time from classification to consultation; therefore, predictive models of the median and 95th percentile waiting times of patients based on queuing theory have been studied [24].

The findings of some studies have revealed better performance of hospital admission prediction models by including text data. A study was conducted to train a deep neural network (DNN) using unstructured text data from EHR datasets of pediatric emergency patients. The model from this study showed that the DNN achieved a high-performance score of 0.892, which was 2% higher in the area under the curve (AUC) than that of the model not including text data [25]. Additionally, LR trained by NLP on the reason for a patient’s visit demonstrated a 2% higher performance score at 0.846 compared to when text data was not included [26].

While acknowledging the considerable value of previous studies on mitigating overcrowding in EDs, limitations remain in the practical implementation of these studies. This is primarily due to a lack of intuitiveness in supporting ED physicians and effectively applying these models in real ED scenarios for patient communication [8, 21–26]. Therefore, we have improved on the existing literature by developing our approach based on these previous studies.

## Methods

### Materials

#### Study design and setting

**Fig. 2:**
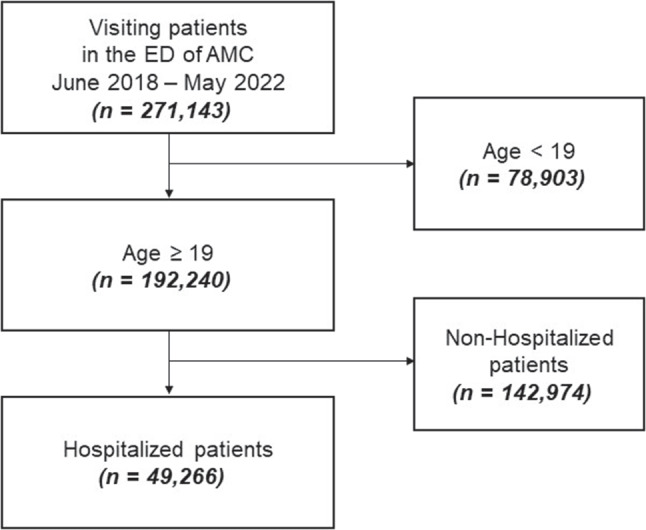
The number of patients who consulted at the ED between June 2018 and May 2022, requiring hospitalization for further care, is depicted. Patients< 19 years old and those who did not require hospitalization were excluded from the final data analyses

This retrospective, single-center, cohort study included a total of 271,143 patients who consulted at the hospital’s ED between June 2018 and May 2022. Data was collected from 192,240 patients ≥ 19-years-old. Of these, 49,266 patients required hospitalization. The exclusion of 142,974 patients from the dataset was due to the difficulty in distinguishing between patients with prolonged waiting times and those who were transferred back to the ED based on the discretion of the physician. Patients who were transferred back to the ED had to wait for hospitalization and frequently experienced a total waiting time exceeding 24 hours, which made them ineligible for our study. Although this exclusion could have introduced bias to our results, the decision was made because it was challenging to distinguish the reasons for transfer in the dataset. This is depicted in Fig. 2.

This study used ethically pre-approved data and underwent Asan Medical Center (AMC) Institutional Review Board (IRB) review (IRB 2021-0321), which was conducted by the Declaration of Helsinki (2008). De-identified data were extracted from the “data” and “clinical research data” warehouses.

**Fig. 3:**
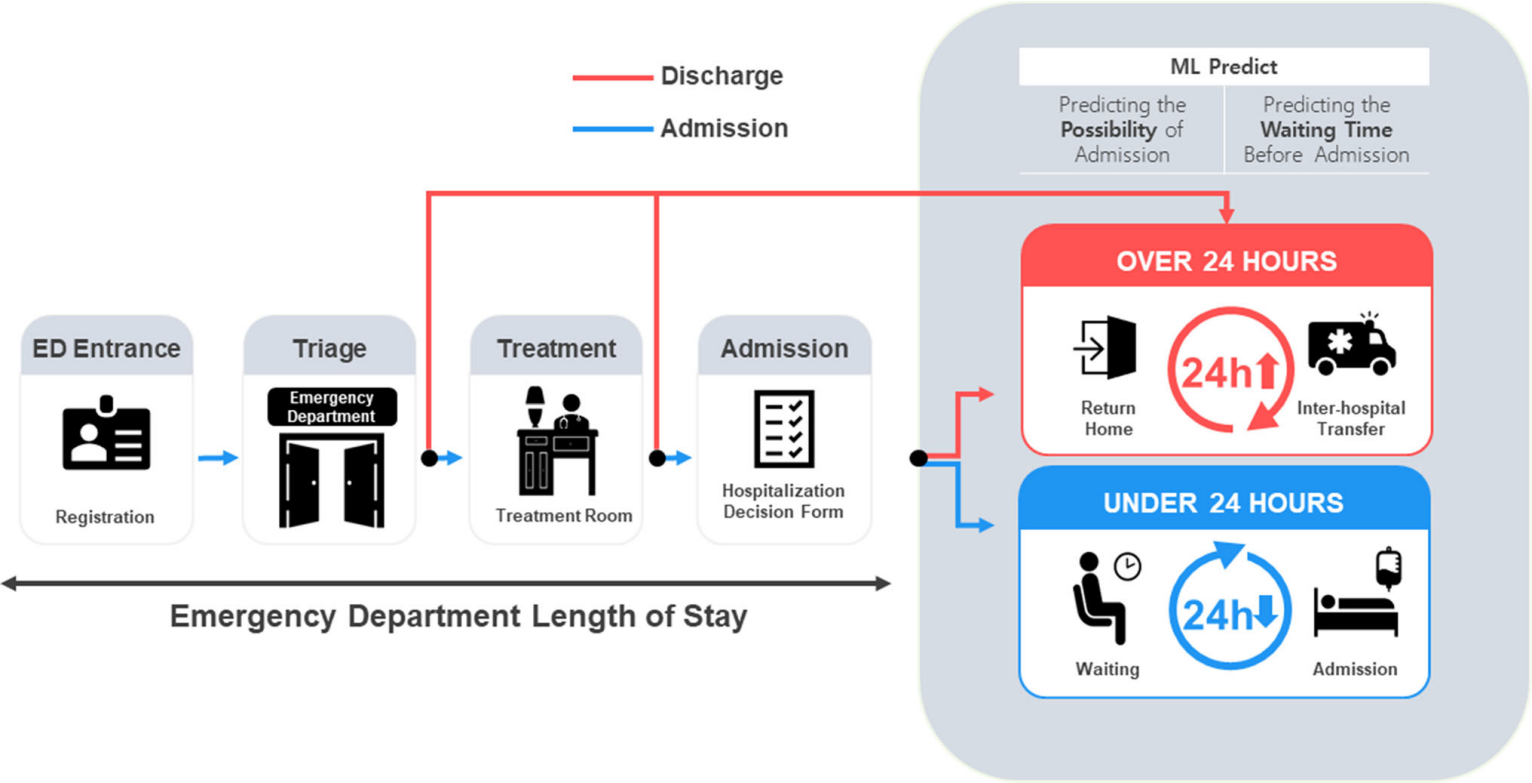
This model of prediction flow is based on the process from ED registration to hospitalization. The red line represents patients who have been discharged from the ED, while the blue line represents hospitalized patients. Depending on the discretion of the ED physicians, decisions regarding discharge can be made in “Triage” and “Treatment”. The ED length of stay is defined as the waiting time from the arrival of the patients at the ED to when they are discharged from the ED. According to the prediction of the ML model, a patient who has received a hospitalization decision form will be classified as “discharged” if the total waiting time exceeds 24 hours or classified as “hospitalized” if it does not

#### The process from ED registration to inpatient hospitalization

As per the government guidelines, the ED length of stay should be ≤24 hours after arrival at our hospital in Seoul, South Korea. Upon arrival at the ED, patients first undergo registration and then proceed to the hospital office to obtain the ED treatment application form. Thereafter, they move to the triage area to undergo a preliminary medical examination and are subsequently assigned to a treatment room based on the severity of their condition. After the patients have submitted their medical receipts to the ED nurses in the treatment area, the ED physicians will review the recommended treatment. Depending on the severity of the condition or the appropriateness of hospitalization, patients are discharged from the ED. If the patient requires hospitalization for treatment, a hospitalization decision form is prepared. Subsequently, the patients are admitted on the designated admission date stated on the hospitalization decision form. The input variables we use for prediction are based on this setup, allowing immediate predictions afterward. The waiting time in the ED refers to the period from ED registration until receipt of the hospitalization decision form, with a discharge from the ED. The models we have developed predict the likelihood of hospitalization and estimate the waiting time, with a borderline within 24 hours of the patients presenting at the ED. Depending on the likelihood of admission, if the expected waiting time exceeds 24 hours, the patient may be advised to be transferred or discharged. This process is shown in Fig. 3.

#### Description of independent variables

Demographic, administrative, and clinical variables were collected from the information provided during the process of the ED visit registration to the hospitalization decision form. A detailed presentation of the variables generated for each category of data in Table S1 is depicted.

### Data pre-processing

#### Pre-processing of structured data

Table S2 describes and lists the main structured variables of this study. Duplicate or “Admission” tagged features were removed because they were classified as future variables and were unnecessary for this study. To calculate the waiting time, we created a variable that subtracted the ED visit time from the admission reservation time and converted it into hourly units. Text-type features were converted into integers. For example, ‘4.0’ indicated the ED location code was converted to the number “4.” Missing value transformations were determined based on the data type. Variables were populated with “Unknown,” “U,” or “0.” The target value, which is the time spent in the ED, was expressed as a decimal by converting minutes into hours.

#### Pre-processing of unstructured text data

Variables with unstructured text included “Isolation types” and “ED Sickbed Information Notes.” Each text variable was pre-processed through normalization, tokenization, word frequency counting, term frequency-inverse document frequency (TF-IDF), and missing value filling. First, after filling in the missing values with “Unknown,” unnecessary words, such as special characters and numbers, were removed. Additionally, after consolidation, all letters were changed to lowercase [27]. Second, for tokenization, the length was cropped to 2-5 words for word extraction. Korean words that were not automatically tokenized were manually spaced [27]. Table S3 shows the original and modified text through normalization and tokenization. Third, the tokenized words were converted into bag-of-words-encoded vectors. In the “Isolation type” column, only words with a document frequency (DF) of ≥ 10 and “Sickbed Information Notes” of ≥ 500 were extracted. The df number criterion was set based on the interval in which the frequency of tokens generated for each column sharply differed. Fourth, we applied TF-IDF, which calculates the importance of words in a document-term matrix (DTM) [27]. This technique assigns weights to specific words in a DTM by applying a specific formula to the word and document frequency. It is simple to use and characteristically performs better in ML models. The TF-IDF score created a DTM. Thereafter, the value was obtained by multiplying TF, which represented the relative frequency of term t in document d, and IDF measured the importance of the term t in the corpus. This can be expressed in the equation as follows:

$$\begin{aligned} TF(t,d)= & {} \frac{\textit{Number of occurrences of term t in document d}}{\textit{Total number of terms in the document d}}\\ IDF(t,D)= & {} \frac{\textit{Total number of documents in the corpus}}{\textit{Number of documents with term t in them}}\\ TF-IDF= & {} TF(t,d) \times IDF(t,D) \end{aligned}$$

Finally, the TF-IDF scores for each selected word were transformed into a two-dimensional array. Rows processed as missing values at this time because they did not contain words were replaced with 0.0. The variables generated through this process were assigned new names, as indicated in Table S4.

#### Target variable

**Fig. 4:**
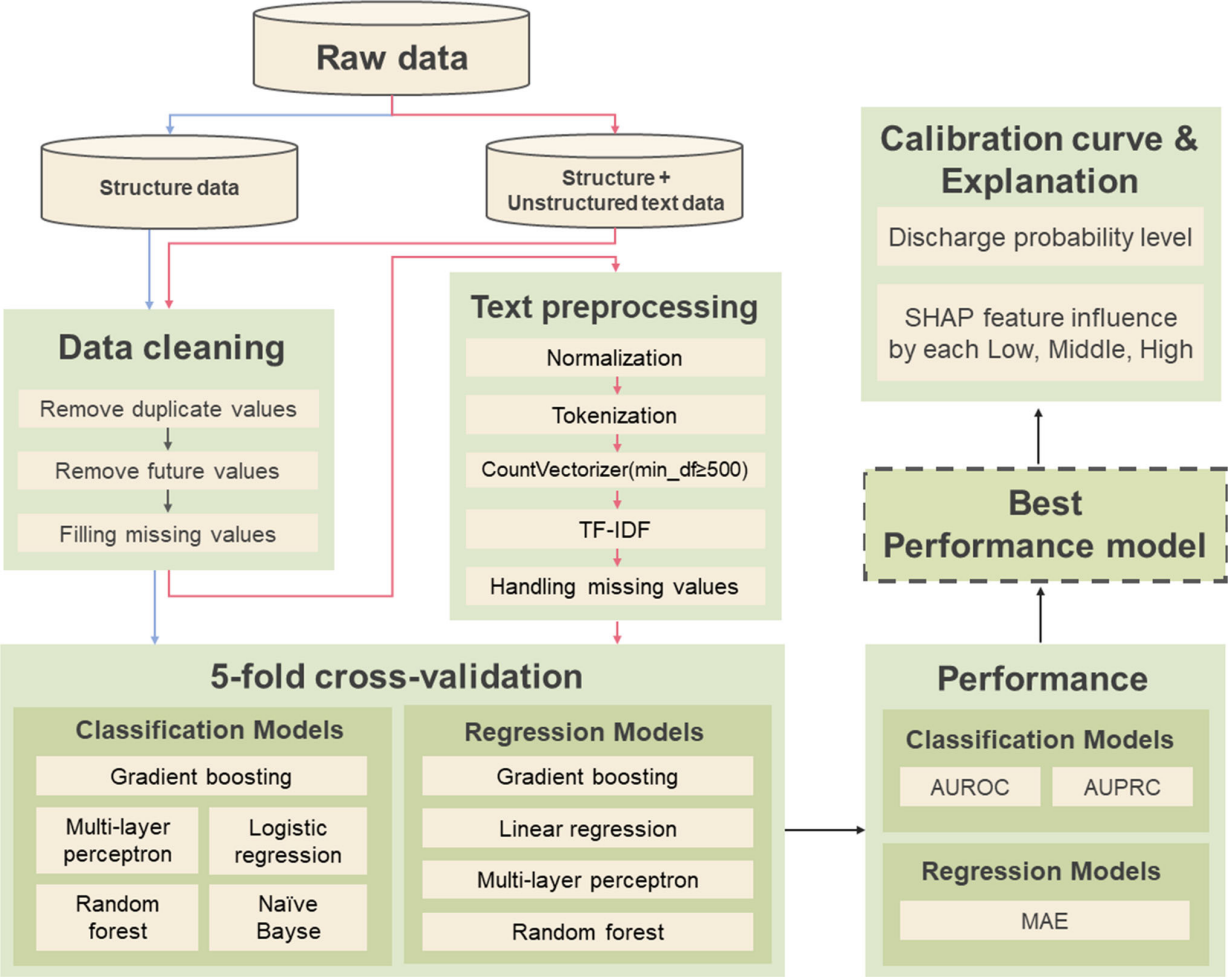
The overall flow of the prediction method and XAI usage in this study are depicted

The “target value” of the waiting time prediction model is a numerical value denoting the waiting time. For the classification model regarding the likelihood of hospitalization in ED, the target value was standardized as 24 when it exceeded 24 hours for training. If the actual waiting time was < 24 hours, it was converted to the number “0”. If it was ≥ 24 hours, it was converted to the number “1”. The result value was set in binary format. Finally, a total of 82 variables were used, including 61 structured data variables and 21 variables generated by NLP from unstructured text data. Figure 4 depicts the overall flow of the prediction method and usage of XAI in this study.

### Admission prediction ML-based models

In this study, two ML approaches were applied based on the research objectives. First, the ML classification model was used to predict the likelihood of hospitalization for patients waiting in the ED. Second, an ML regression model was used to predict the waiting time until hospitalization.

We evaluated five different ML models to predict the likelihood of hospitalization. These included the Gradient Boosting Machine (GBM) [28] and Extreme Gradient Boosting (XGB) [29] models. Typically, XGB aims to improve models by sequentially and iteratively training multiple decision trees on training data. It minimizes prediction errors and avoids overfitting through regularization techniques, ultimately improving generalization performance. Random Forest (RF) [30] is an ensemble learning algorithm based on decision trees, where multiple decision trees are combined to create a single model. During the construction of decision trees, features and data are randomly selected. Additionally, the prediction results are aggregated to prevent overfitting and achieve stable predictions. Multi-layer perceptron (MLP) [31] is a neural network architecture consisting of multiple hidden layers. MLP is capable of modeling non-linear correlations, making it suitable for addressing complex problems. The weight updates using backpropagation enable learning from diverse inputs and outputs. LR is a statistical technique [32] that models the correlation between inputs and outputs. It involves taking a linear combination of input features and weights, passing it through a logistic function to generate probability values between 0 and 1, and using a threshold to predict classes. LR is simple and interpretable; however, it is limited in modeling non-linear correlations among input features. Naïve Bayes (NB) is a supervised learning algorithm based on the principles of Bayes’ theorem. This model assumes that input variables are independent and directly impact only one output variable, making it computationally efficient and quick to train [33]. Nonetheless, in many real-world scenarios, it may be unrealistic to assume such independence among input variables. This limitation can restrict the practical applicability of the model.

We used XGB, RF, MLP, and LR [34] among the ensemble techniques to determine the waiting time until hospitalization. Moreover, among the five classification models described earlier, XGB, RF, and MLP can be used for regression tasks. Thus, the same models were used to streamline the training process. Additionally, LR is a method used to model correlations between two or more variables, assuming the correlations between the variables are linear. Therefore, changes in one variable are proportionally related to changes in other variables. LR creates a prediction line based on these suppositions, making it simple and easy to interpret. However, with real-world data, complex non-linear correlations often exist, making it difficult to model.

The performance of these models was compared by training them on both structured and unstructured text data. Finally, we used XGB which resulted in it being the model with the best performance. For the hyperparameters, a grid search was used for fine-tuning.

### Cross-validation

Between June 2018 and May 2022, a total of 49,266 patients who consulted at the hospital’s ED in Seoul, South Korea, required hospitalization. Of these, 44,753 patients (90.8%) were admitted within 24 hours, while the remaining 4,513 patients (9.2%) were not admitted within 24 hours. Despite the imbalance in the data classes, our goal was to use 5-fold cross-validation without shuffling the data to assess the predictive performance of the admission prediction model on new patient data in the future. Data bias is conventionally reduced by 5-fold cross-validation in ML model evaluation, thus improving accuracy [35]. Five cross-validations were performed by dividing 44,753 patient data into five equal parts, with 80% of the data used for training and 20% used for validation.

#### Data transformation

We transformed the data at every fold validation for model training. The data were divided into categorical and numeric types based on variables. For the categorical type, the target encoder had to be applied. This encoding method is characterized by expressing the correlations between similar categories. However, this correlation is limited to only categories and targets. The advantage of this method is that it facilitates fast learning without compromising on the quantity of data; consequently, we implemented it in our model. To achieve balance, the mean was considered by setting the smoothing to 1.0 and minimum samples to 1. Furthermore, any columns with zero variance had to be dropped. For numeric types, a MinMaxScaler was used. This ensured that the value of each variable adhered to a specific range or rule; here, the data was converted to a range between 0 and 1.

#### Performance

The predictive performance of the ED hospitalization model was evaluated using the area under the receiver operating characteristic (AUROC) and the area under the precision-recall curve (AUPRC). The receiver operating characteristic (ROC) curve was used to indicate the performance of a classification model when determining a patient’s hospitalization status that was represented in binary form as positive or negative. The X-axis represented the false positive rate (FPR), and the Y-axis represented the true positive rate (TPR) with a proportional correlation between the two. Measuring the AUROC was used to evaluate performance. The closer this value was to 1, the better the model and the higher the likelihood of hospitalization. The AUPRC was indicated by setting the X-axis to recall and the Y-axis to precision. These two values are inversely proportional to each other and form a downward curve towards the right. Both precision and recall are ideal models when close to 1; therefore, the closer the value of AUPRC is to 1, the better the model. The performance of the predictive waiting time model for patients consulting at the ED was evaluated using the mean absolute error (MAE). MAE is the average of the absolute sum of the differences between the predicted waiting time by the regression model and the actual time. The error size was accordingly reflected. Because MAE is a highly sensitive indicator, the difference between the predicted ED waiting times by the model can be recognized immediately. In the given formula, $e_{i}$ represented the difference between the actual value, $y_{i}$, and the predicted value, $ypred_{i}$. MAE was calculated as the average of the absolute differences divided by the total number of data points:

1 $$\begin{aligned} MAE= & {} \frac{1}{n}\sum _{i=1}^{n}\left| e_{i} \right| \end{aligned}$$

2 $$\begin{aligned} * e_{i}= & {} y_{i} - ypred_{i} \end{aligned}$$

### Calibration curve and SHapley Additive exPlanations

We utilized a calibration curve to identify the factors that impacted the determination of discharged patients in general ED scenarios. Additionally, we employed SHapley Additive exPlanations (SHAP) for XAI. The calibration curve revealed the realistic predictive correlation between the predicted and actual probability of admission likelihood. Thus, if the patient’s admission probability was 80%, it indicated that the actual admission probability was also 80%. We used the calibration curve of XGB with text data that had the highest performance among the models for predicting hospitalization. The X-axis of the calibration plot of this model was divided by 0.1 units. After calculating each section’s event rate, the predicted and actual probabilities were displayed as a bar plot. Moreover, the proportion of non-hospitalized patients was similarly calculated and divided into three categories: Low (0-0.5), Medium (0.6-0.8), and High (0.9-1.0). The criteria for dividing the levels were based on the proportion of patients who were not admitted within 24 hours at each interval. The classes Low, Middle, and High comprised 1017, 173, and 245 patients, respectively.

SHAP is a technique that transparently reveals the internal functioning of a complex AI model. A Shapley value is calculated by measuring the average change in the presence or absence of a feature when combining multiple features, assisting in distinguishing the importance of each individual feature. Additionally, the importance of each feature can be calculated using the feature importance function in tree-based or boosting algorithms. However, this technique, used to identify variables, affects the prediction through permutation. Due to the estimation limit imposed by the degree of error, the importance of variables may vary each time the algorithm is executed, potentially leading to the oversight of dependencies between features. Therefore, models with correlations between features should avoid using the feature importance function. The Shapley value utilizes the concept of independence between variables as a key idea. It is used to calculate the impact of variables by comparing the results obtained when all combinations of variables related to a specific variable are input. The selection is made based on the effect on the target variable. Moreover, it can explain the negative and positive correlations between the value of the result and the variable. The technique should be selected based on the viewpoint. In our study, we chose to use SHAP because there was a high likelihood of a dependency between the features [36].

Each level was applied to the SHAP summary plot and plots bar. The SHAP summary plot can determine the magnitude of a feature based on its Shapley value. The plots bar calculates the global importance by averaging the absolute values of each Shapley value. Consequently, it is possible to grasp the detailed influence of the variable on the model. Among the Shapley values that were generated using the plots bar, the top eight values were depicted using radar plots.

**Table 1:** Evaluation of 5-fold cross-validation for each ML model using AUROC and AUPRC

	RF		MLP		LR		NB		XGB	
	AUROC	AUPRC	AUROC	AUPRC	AUROC	AUPRC	AUROC	AUPRC	AUROC	AUPRC
Fold 1	0.845	0.323	0.864	0.286	0.846	0.225	0.722	0.241	0.882	0.412
Fold 2	0.875	0.395	0.789	0.259	0.785	0.228	0.740	0.201	0.881	0.437
Fold 3	0.862	0.587	0.812	0.482	0.804	0.447	0.779	0.383	0.875	0.604
Fold 4	0.824	0.371	0.792	0.335	0.752	0.206	0.717	0.188	0.837	0.400
Fold 5	0.756	0.472	0.684	0.335	0.755	0.363	0.720	0.362	0.822	0.542
Mean	0.832	0.430	0.788	0.339	0.788	0.294	0.736	0.275	0.860	0.480
(SD)	(0.042)	(0.092)	(0.058)	(0.077)	(0.035)	(0.095)	(0.023)	(0.082)	(0.025)	(0.080)

**Table 2:** Evaluation of 5-fold cross-validation for each ML-model with NLP using AUROC and AUPRC

	RF with NLP		MLP with NLP		LR with NLP		NB with NLP		XGB with NLP	
	AUROC	AUPRC	AUROC	AUPRC	AUROC	AUPRC	AUROC	AUPRC	AUROC	AUPRC
Fold 1	0.891	0.525	0.896	0.474	0.890	0.436	0.759	0.360	0.922	0.577
Fold 2	0.948	0.703	0.936	0.701	0.920	0.609	0.892	0.630	0.953	0.735
Fold 3	0.946	0.780	0.930	0.726	0.932	0.723	0.913	0.759	0.955	0.822
Fold 4	0.897	0.601	0.893	0.575	0.856	0.512	0.799	0.411	0.908	0.634
Fold 5	0.833	0.612	0.835	0.585	0.826	0.558	0.783	0.511	0.875	0.667
Mean	0.902	0.644	0.898	0.612	0.885	0.568	0.829	0.534	0.922	0.687
(SD)	(0.042)	(0.088)	(0.036)	(0.092)	(0.039)	(0.096)	(0.061)	(0.145)	(0.030)	(0.085)

## Results

### Performance of the ML-based predictive models

**Fig. 5:**
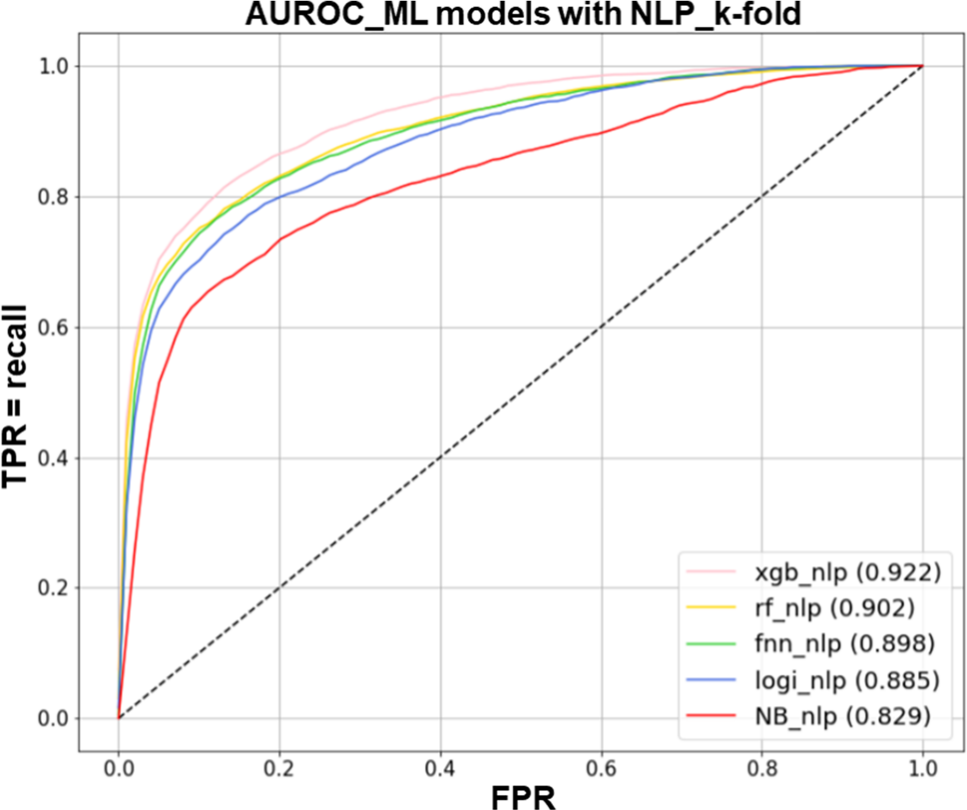
AUROC curves of hospital admission prediction models with NLP

**Fig. 6:**
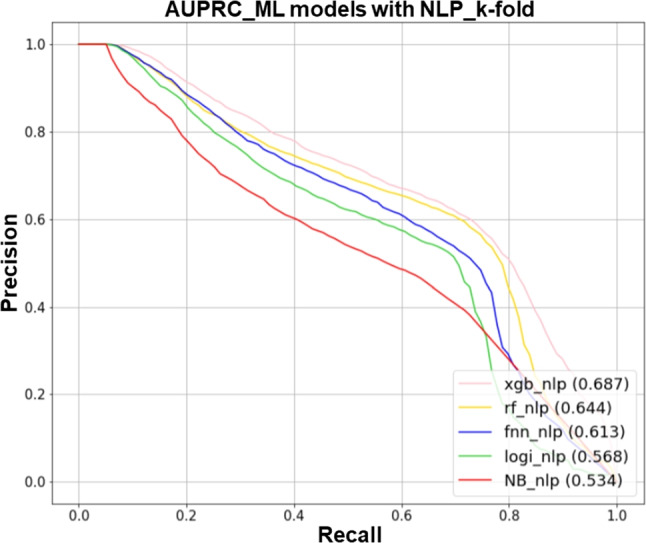
AUPRC curves of hospital admission prediction models with NLP

Tables 1 and 2 show the AUROC and AUPRC metrics for each fold of a 5-fold cross-validation of ML models that have been trained to predict the likelihood of hospitalization within 24 hours after arrival at the ED. Comparing the two models, the models trained with unstructured text data outperformed those without unstructured text data by 6-10% and 20-28% on AUROC and AUPRC, respectively. The average values of AUROC and AUPRC for XGB, including text data, were the highest compared to other classification models. The AUROC and AUPRC were 0.922 (SD 0.030) and 0.687 (SD 0.085), respectively. By presenting the mean AUROC and AUPRC of each classification model using text data in Figs. 5 and 6, XGB evidently exhibited the best performance.

Tables 3 and 4 show the MAE and average values for each fold of the regression ML model that predicts the waiting time from arrival at the ED to hospitalization. The MAE of the XGB model that included text data revealed the smallest difference when compared to that of the other models. The MAE of XGB with NLP revealed a difference of approximately 3.02 from the actual value and equated to 3 hours when converted into time. The difference was 30 minutes less than that of a normal XGB model, equating to 3 hours 30 minutes with a time conversion of 3.53.

We selected the XGB with text data as the final model. XGB has fast learning and classification speed due to parallel processing and strong durability with its overfitting regulation function. Furthermore, it demonstrates excellent predictive performance in both classification and regression tasks; therefore, it was hypothesized to predict the likelihood of hospitalization accurately and expected waiting time as a single model, making the process uncomplicated.

### Variable importance through SHAP

**Fig. 7:**
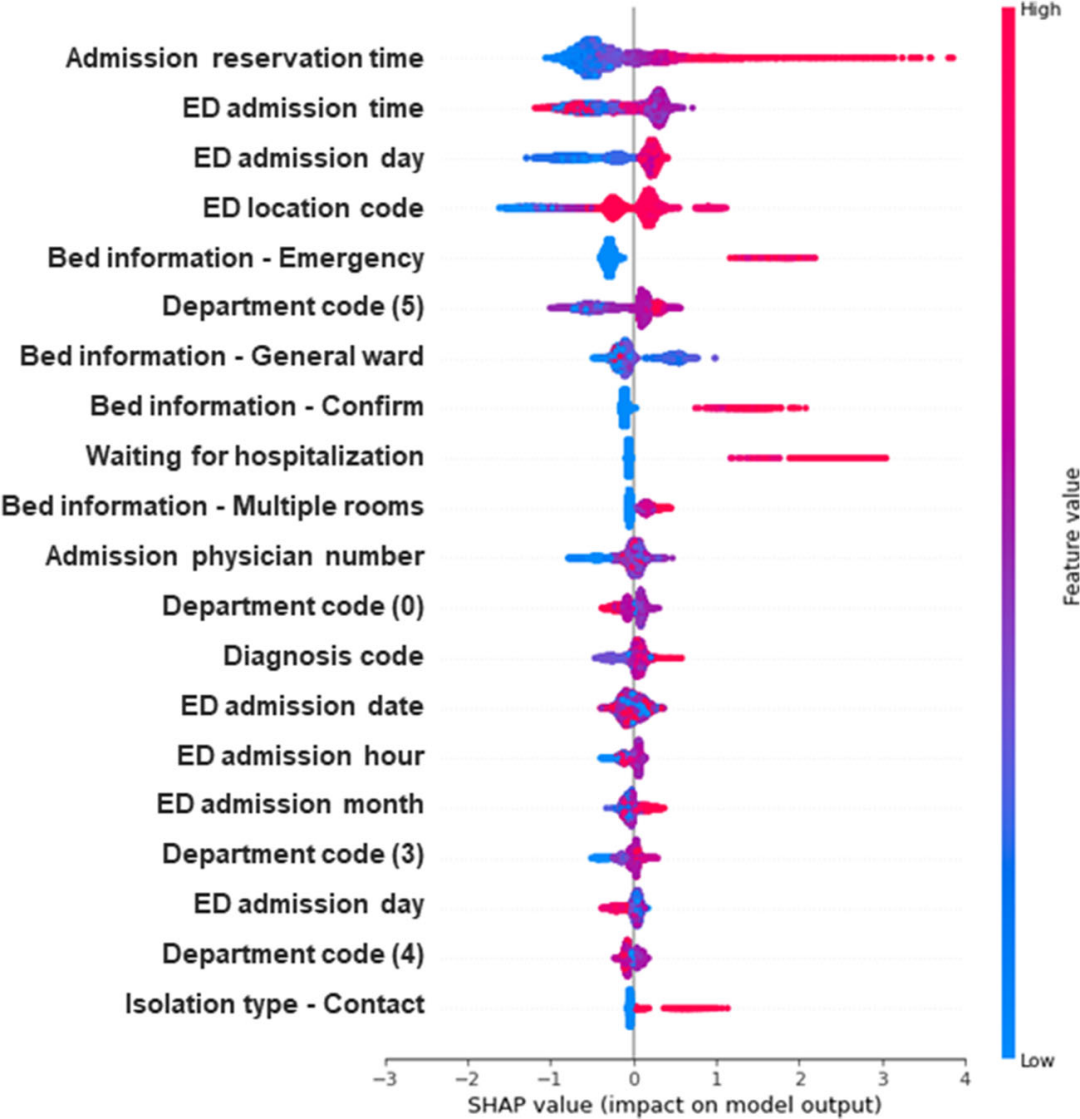
Shapley effect values for variables in the XGB model with text data

The summary_plot of SHAP means that the performance contributing to the model’s prediction increases as it moves up from the bottom of the y-axis. The x-axis represents the magnitude of each variable’s impact on the outcome value. In our study, we found that the color red was associated with a delay in admission within 24 hours, while the color blue was found to have an impact on the admission process. For example, in the case of ‘Bed information - Confirm’, it most likely indicates a low likelihood of hospitalization. Conversely, in the case of ‘ED admission day’, where the blue color appears longer, it can be considered a variable that increases the likelihood of hospitalization within 24 hours. The SHAP results of XGB with the highest performing text data are shown in Fig. 7. Tables S2 and S4 of the Supplementary Information were referred to for a description of the variables in Fig. 7.

The SHAP summary plot demonstrates that the performance contributing to the model’s prediction increases as it moves up from the bottom of the Y-axis. The X-axis represents the magnitude of each variable’s impact on the outcome value. In our study, we found that the color red was correlated with a delay in admission within 24 hours, while the color blue was found to have an impact on the admission process. For example, in the case of the variable “Bed information - Confirm,” a low likelihood of hospitalization was indicated. Conversely, in the case of the variable “ED admission day,” where the blue color appeared longer, the likelihood of hospitalization within 24 hours increased. The SHAP results of XGB with the highest-performing text data are shown in Fig. 7. Tables S2 and S4 are referred to for a description of the variables in Fig. 7.

**Table 3:** Evaluation by MAE of 5-fold cross-validation for each ML-model

	MAE			
	RF	MLP	LR	XGB
Fold 1	3.67	3.43	3.80	3.53
Fold 2	3.55	3.51	3.99	3.55
Fold 3	3.66	3.98	4.25	3.52
Fold 4	3.40	3.35	3.73	3.38
Fold 5	3.87	3.95	4.21	3.66
Mean	3.63	3.64	3.99	3.53
(SD)	(0.154)	(0.267)	(0.209)	(0.090)

**Table 4:** Evaluation by MAE of 5-fold cross-validation for each ML-model with NLP

	MAE			
	RF with NLP	MLP with NLP	LR with NLP	XGB with NLP
Fold 1	3.35	3.00	3.55	3.38
Fold 2	2.66	2.90	3.47	2.77
Fold 3	2.69	2.71	3.45	2.61
Fold 4	3.03	3.00	3.51	2.96
Fold 5	3.54	3.67	4.02	3.35
Mean	3.05	3.05	3.60	3.02
(SD)	(0.349)	(0.325)	(0.213)	(0.310)

**Fig. 8:**
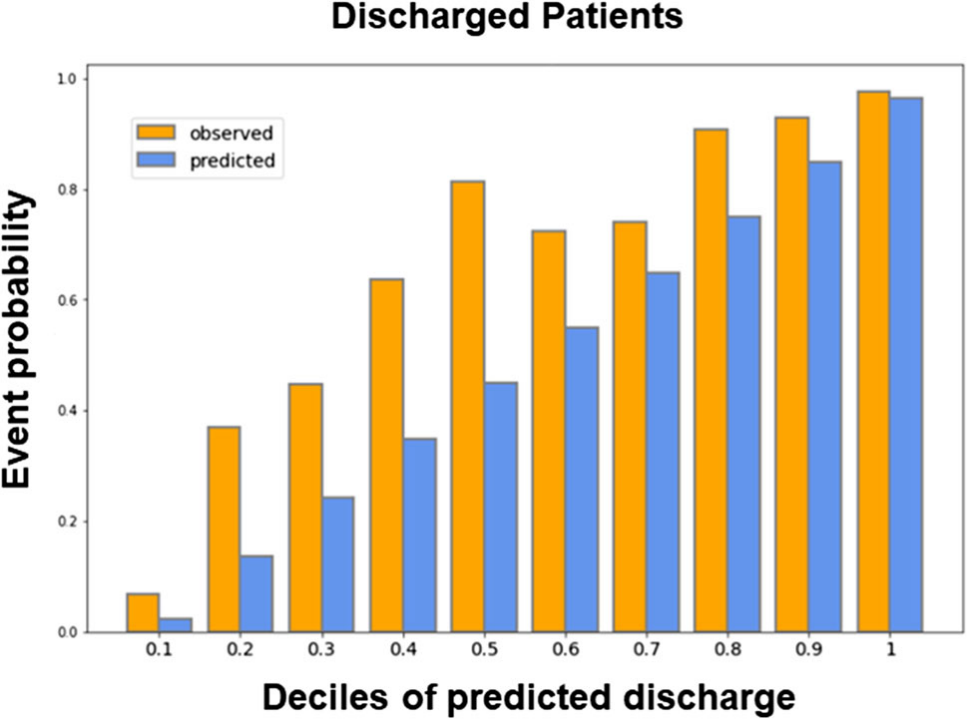
The X-axis is divided into deciles based on increments of 0.1 for the predicted discharge probability of the patients, while the Y-axis represents the actual discharge rate. The orange bar represents the rate of observed discharged patients, while the blue bar represents the rate of predicted discharged patients

We analyzed the calibration curve to determine the statistical significance of the variables affecting patients who either had a low or high probability of being admitted within 24 hours of their arrival at the ED. In addition, the XGB model with the best performance was used to analyze the target value, set to “1,” of patients who were not hospitalized within 24 hours. The X-axis of the calibration curve was delineated as the average value, established as the predict_proba of the predicted value. The Y-axis delineated the average of the actual values (the fraction of positives correctly classified). By creating a histogram of the X- and Y-axes of the calibration curve, the ratio between the predicted value and the actual value was plotted, as presented in Fig. 8. As revealed in the histogram, the likelihood of events for observed discharged patients was higher than that of predicted discharged patients.

**Fig. 9:**
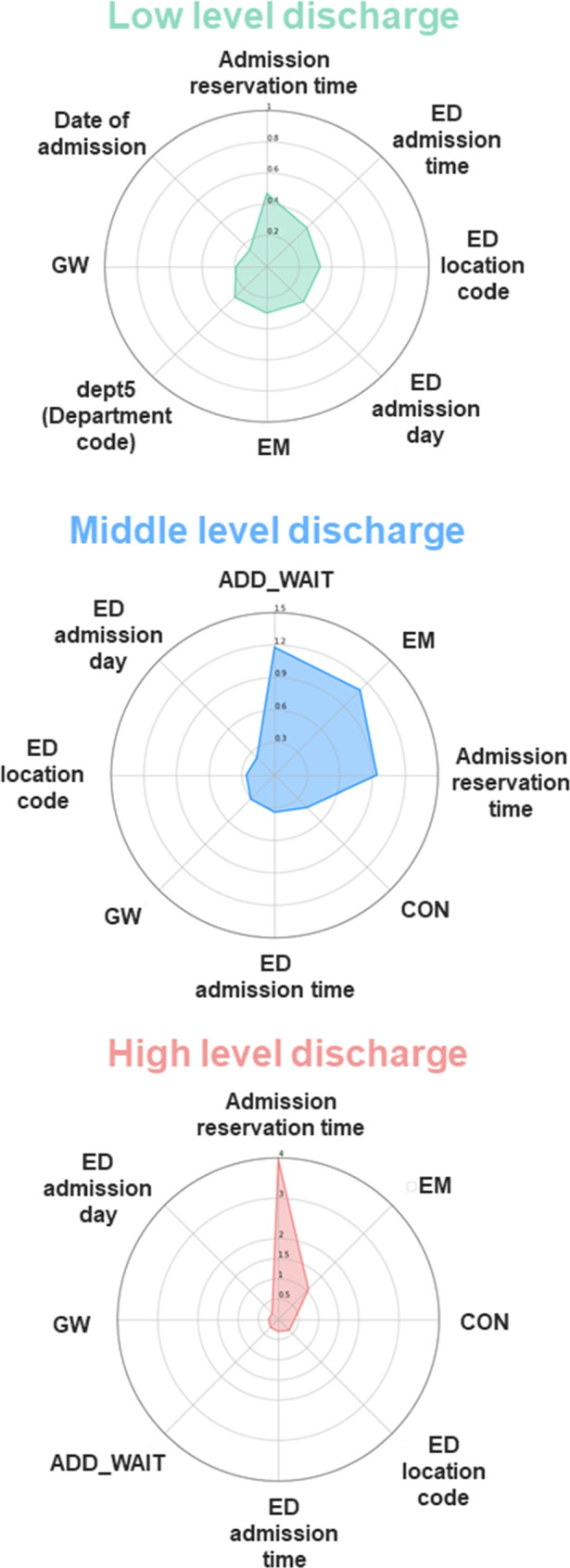
Radar plots of the influence of Shapley values of the top eight variables by Low, Middle, and High are shown. Green is Low, Blue is Middle, and Red is High

The number of patients corresponding to each level was 10 186, 221, and 255 for the Low, Middle, and High level classes, respectively. Each level was confirmed by SHAP. We replaced the top eight quantifiable variables in the bar plots with radar plots, as depicted in Fig. 9. For the Low level class, “Admission reservation time” was the highest with a value of 0.47, followed by values for the “ED admission time” (0.35), “ED location code”(0.33), “ED admission day” (0.32), “EM” (0.3), “dept5” (0.28), “GW” (0.19), and “Date of admission” (0.15). For the Middle level class, “ADD_WAIT” was the highest with a value of 1.18, followed by “EM” (1.11), “Admission reservation time” (0.94), “CON” (0.42), “ED admission time” (0.34), “GW”(0.31), “ED location code” (0.26), and “ED admission day”(0.23). For the High level class, “Admission reservation time” was the highest with a value of 3.95, followed by values for the “EM” (1.06), “CON”(0.4), “ED location code”(0.38), “ED admission time” (0.3), “ADD_WAIT” (0.28), “GW” (0.24), and “ED admission day” (0.22). Descriptions of the variables are mentioned in Tables S2 and S4.

With these results, it is possible to demonstrate to the patient the likelihood of being admitted and inform them about their expected waiting time. If admission is not possible, the patient can be informed of the reason. As a result, EDs and hospitals will exhibit high operational efficiencies by implementing these methods.

## Discussion

The full dataset for our study utilized patient information obtained between June 2018 and May 2022 from a hospital in Seoul, South Korea, where patients were admitted through the ED. Moreover, this dataset included a specific subset of patients who were hospitalized in a designated COVID-19 environment. In addition to the economy and society, the recent global pandemic, COVID-19, has impacted the ED. After the outbreak of COVID-19, the waiting time in the ED has become irregular, as patients are only allowed to visit the hospital once their polymerase chain reaction (PCR) test results are confirmed in order to prevent the spread of infectious diseases. Furthermore, hospitals have experienced shortages of wards, as they have had to allocate infected and non-infected patients to separate wards [37]. Nationally, South Korea has established a centralized funding system [38], and this system offers patients considerable autonomy. Patients can consult at primary and secondary hospitals, as well as the ED, without requiring a referral. Additionally, if patients receive an initial diagnosis from a physician, they can consult at tertiary hospitals with a referral form issued by the diagnosing physician. Moreover, the government supports cost-sharing programs that assist in reducing the financial burden on patients during hospital consultations [38, 39]. In contrast, the healthcare workforce regarding medical personnel remains below the average, as advised by the Organisation for Economic Co-operation and Development (OECD) [40]. This shortage of medical staff leads to inadequate patient care that is further exacerbated by the challenge of managing a large number of patients.

We compared ML-based models trained on data during this period. XGB revealed a high performance with an AUROC of 0.860 and an MAE of 3 hours 30 minutes. Additionally, we trained unstructured text data on XGB to predict the likelihood of hospitalization. Our model achieved an AUROC score of 0.922 and an MAE of 3 hours, demonstrating strong performance. Our model had an AUROC that was 6% higher and a MAE that was 30 minutes less than the existing XGB. Regarding text manually entered by doctors and nurses in the ED, such as ED sickbed information notes, this contains information that can be immediately updated based on new environmental changes, including COVID-19. This differed from simple computer-entered demographic, administrative, and clinical variables.

The text data contained important information regarding the patient’s condition or hospitalization [12]. We applied TF-IDF to this data and further trained it on the existing XGB to exhibit improved performance in predicting hospitalization and estimated waiting time. By applying SHAP to the XGB model with added text data, we revealed the contribution of features based on the probability level of discharged patients. Informing the patient about the reason for not being hospitalized involves clearly counseling the patient about the condition. This practice enables the efficiency and accuracy of emergency physicians, ultimately reducing overcrowding in the ED. The common variable that showed the greatest effect across the Low-, Middle-, and High-level classes was the “Admission reservation time.” In the High level class, with the least likelihood of hospitalization, the effects of the parameters gradually decreased in the order of “EM,” “CON,” and “ED location code.” By utilizing this information to enhance the process in the healthcare system, hospitals can appropriately elevate patient admission rates and decrease waiting times.

The study’s findings revealed that the variables had a statistically significant influence on the results, as demonstrated by SHAP. These findings can improve the efficiency of the operation of the ED by enhancing the processes related to variables in unpredictable situations, such as COVID-19. High-performance models and the identification of adjustable variables can facilitate the maintenance of ML models and augment existing models. Our model is a useful and inexpensive tool that provides decisive clinical support to ED physicians constructed on readily available patient medical data.

Despite this considerable performance, the estimated waiting time provided by the ED to the patient and the time perceived by the patient may be different. These differences should be resolved through effective communication with patients in the hospital. Additionally, the ED should create a comfortable waiting room environment and provide feedback on estimated waiting times, notifying the patient of any updates in progress. Furthermore, it is important to regularly check the waiting room area and apply a personalized approach to patient care. If these series of improvements are integrated, the overcrowding of the ED will be reduced [41–43].

Several limitations should be considered in this study. First is the limitation regarding the objective evaluation of the clinical usability and acceptability. It may be challenging to empirically appraise the actual value of the application of results of this independent single-center study for other hospitals and clinicians. However, this study was initiated in response to a request from physicians working in the ED to address overcrowding issues. To make the algorithms more user-friendly for practical use in the ED, we developed ML models that provided predictive results and worked on software development. Furthermore, a systematic evaluation of the effectiveness of the EMR system is planned.

The second limitation is the lack of validation for external generalizability. Our models can be applied to predict the likelihood of hospitalization and waiting time in the ED of tertiary hospitals in Korea. However, since each hospital has different systems, users of models may experience the inconvenience of having to learn new functionalities. Additionally, the EMR systems utilized by each hospital have varying interfaces and terminologies for input variables. This discrepancy may lead to reduced accuracy when comparing the models of our study to other models if they are implemented without modifications. Although validation is possible for commonly used variables, differences in variable definitions could impact accuracy. Nevertheless, this research was initiated in response to clinical requests from hospitals, indicating a high probability of its relevance in other hospitals.

The third limitation of developing prediction models for hospital admissions in the ED of Korean hospitals is the data imbalance caused by including mixed data from before and after the outbreak of COVID-19. This limitation may restrict the performance of the models of this study. However, according to the policies of the Korean government and hospitals, the ED needs to be sufficiently flexible to accommodate various diseases that are unrelated to COVID-19. Thus, a system has been implemented that requires COVID-19-suspected patients to be admitted after PCR testing. Moreover, numerous changes have been made to the ED system in response to the COVID-19 outbreak. In this context, we believe it is appropriate to use mixed data, including data related to COVID-19, for predicting sudden changes in the system that we aim to address. Therefore, we value the high performance of our study results. This study proposed prediction models for situational changes that may occur in the healthcare system due to various disease occurrences, including COVID-19.

The fourth limitation is regarding the sole use of XAI which does not suffice when counseling patients on non-admission. While XAI offers interpretable features, it only contributes individual variables to specific predictions, making it challenging to fully comprehend the overall behavior of the model. Consequently, particularly if a specific variable has a substantial impact on non-admission, the explanation given to the patient would still be inadequate. Moreover, XAI relies on the trained model and specific data distributions, thus making it arduous when generalizing SHAP values once new data is introduced. Exclusively relying on SHAP values to drive changes in specific areas of the ED is limiting. However, ED physicians can still provide patients with appropriate explanations regarding non-admission using this functionality, supporting the counseling of patients.

Despite these limitations, we have developed a high-performance predictive model. This is the first time that we identified the contribution of variables by dividing patients who were not hospitalized by levels.

## Conclusion

We created models that included unstructured text data that can be easily overlooked to predict the likelihood of hospitalization within 24 hours and estimated waiting times for admission for patients using the ED. The data consisted of demographic, administrative, and clinical information that was easily obtained from registration at the ED until admission to the hospital. The XGB with text data model predicts the likelihood of admission and the expected waiting time within 24 hours of an ED length of stay. Moreover, via SHAP it includes a function that identifies the variables that have the greatest impact on difficult admissions. It has been confirmed that applying NLP to unstructured text data has a considerable effect on the target.

This model will contribute to improving the accuracy and speed of decision-making for ED physicians. The influence of variables on the number of patients who are not admitted within 24 hours, as identified through XAI, provides crucial information for ED physicians to explain to patients who cannot be admitted under the “24-hour Emergency Department Restriction Act.” By utilizing this information, the estimated average length of stay for all patients can be determined by considering the estimated waiting times. This lets doctors promptly notify patients about non-admission and transfer options, thus optimizing hospital operational costs and reducing waiting times. Furthermore, hospital managers can obtain information to optimize waiting times, improve hospital processes, and minimize operational costs. Such information greatly improves hospital operations and economic efficiency and can be particularly useful in mitigating ED overcrowding. After implementing this model in a real ED, it is crucial to collect feedback from healthcare professionals and consistently update and optimize the model’s performance by incorporating new data and receiving continuous feedback. The model should incorporate real-time patient monitoring to instantaneously integrate the patient’s status into the model. Furthermore, utilizing a customized model that considers individual circumstances and medical conditions can improve the accuracy of admission predictions. These suggestions should be followed to further advance the future scope of research. In the future, if this model is applied and utilized within a monitoring system, such as a command center [13], it is postulated that hospital processes will be greatly improved.

### Supplementary Information

Below is the link to the electronic supplementary material.

Supplementary file 1 (docx 29 KB)

## Data Availability

The datasets analyzed during the current study are not publicly available due to institutional policy but are available from the corresponding author on reasonable request.

## Abbreviations

AI: Artificial intelligence
AMC: Asan Medical Center
AUC: Area under the curve
AUPRC: Area under the precision-recall curve
AUROC: Area under the receiver operating characteristic
DF: Document frequency
DNN: Deep neural network
DTM: Document-period matrix
ED: Emergency department
EHR: Electronic health records
EMR: Electronic medical records
FP: False positive
GBM: Gradient boosting model
IRB: Institutional Review Board
KTAS: Korea Triage and Acuity Scale
LR: Logistic regression
MAE: Mean absolute error
ML: Machine learning
MLP: Multi-layer perceptron
NB: Naïve bayes
NEDIS: National Emergency Department Information System
NLP: Natural language processing
OECD: Organisation for Economic Co-operation and Development
RF: Random forest
ROC: Receiver operating characteristic curve
SHAP: SHapley Additive exPlanations
TF-IDF: Term frequency-inverse document frequency
TP: True positive
XAI: Explainable artificial intelligence
XGB: Extreme gradient boosting
