# Correction to: Prediction of hospitalization and waiting time within 24 h of emergency department patients with unstructured text data

**DOI:** 10.1007/s10729-023-09662-3

**Published:** 2024-01-10

**Authors:** Hyeram Seo, Imjin Ahn, Hansle Gwon, Hee Jun Kang, Yunha Kim, Ha Na Cho, Heejung Choi, Minkyoung Kim, Jiye Han, Gaeun Kee, Seohyun Park, Dong-Woo Seo, Tae Joon Jun, Young-Hak Kim

**Affiliations:** 1 Department of Medical Science, Asan Medical Institute of Convergence Science and Technology, Asan Medical Center, University of Ulsan College of Medicine, 88, Olympicro 43gil, 05505 Seoul, Songpagu Korea; 2 Division of Cardiology, Department of Information Medicine, Asan Medical Center, University of Ulsan College of Medicine, 88, Olympicro 43gil, 05505 Seoul, Songpagu Korea; 3 Department of Emergency Medicine, Asan Medical Center, University of Ulsan College of Medicine, 88, Olympicro 43gil, 05505, Songpagu Seoul, Korea; 4 Big Data Research Center, Asan Institute for Life Sciences, Asan Medical Center, 88, Olympicro 43gil, 05505, Songpagu Seoul, Korea

**Correction to: Health Care Management Science**

10.1007/s10729-023-09660-5

In the originally published version of this article, several occurrences of typographical errors have been spotted by the author and editor in the main text including Figure 8. The following are the list of corrected errors:

1. In the Abbreviations section, a new abbreviation (AMC: Asan Medical Center) has been added.
2. In Section 3.1.1, the text 'This study used ethically pre-approved data and underwent (blind) Institutional Review Board (IRB) review (IRB 2021-0321)...' have been replaced with 'This study used ethically pre-approved data and underwent Asan Medical Center (AMC) Institutional Review Board (IRB) review (IRB 2021-0321)...'.
3. In Section 3.2.3, the text 'If the actual waiting time was ¡ 24 hours, it was converted to the number “0”...' have been replaced with 'If the actual waiting time was < 24 hours, it was converted to the number “0”...'.
4. In Figure 8, the x-axis label was changed from 'Diciles of predicted discharge' into 'Deciles of predicted discharge'.
5. The acknowledgements section has been updated.
6. The 'Ethics approval and consent to participate' statement has been updated.

The original article has been corrected.

