# Supplementary material for: Prediction of hospitalization and waiting time within 24 hours of emergency department patients with unstructured text data
Source: Health Care Manag Sci. 2023 Nov 3;27(1):114–29. doi: 10.1007/s10729-023-09660-5 (PMC10896961; doi:10.1007/s10729-023-09660-5)
Supplement: Supplementary file 1 — (docx 29 KB) [file 10729_2023_9660_MOESM1_ESM.docx]

**Prediction of hospitalization and waiting time within 24 hours**

**of emergency department patients with unstructured text data**

Table S1. Independent variables description

| **Category** | **Data source** | **Description** |
| --- | --- | --- |
| **Administrative** | Non-Hospitalized patients | A patient transferred to another hospital after visiting the ED |
|  | When ED registration | Year, Month, Date, Day, Time, Hour, and Minute |
|  | Hospitalization decision form | Admission order date, Expected date of admission, Scheduled surgery date, Diagnosis code in hospital,  Reason for exceeded schedule,  Tuberculosis ward admission possible, Organ transplant,  CP application, Isolation, Single room availability,  Withholding the hospitalization decision form,  Suspension of patient treatment,  Single room availability at the time of admission reservation, Exceeded schedule, The time from the moment patients visit the ED to a decision for admission |
|  | In emergency department | Pregnant, Severe emergency disease,  Admission decision check, Delay in treatment,  Delay in treatment decision, Waiting for admission, Inspection, Reading, Follow-up, Cross-consultation, Hospitalization decision, Check-out,  Cardio-pulmonary resuscitation (CPR),  Do not resuscitate (DNR), Intensive care unit (ICU),  Multiple traumas, Emergency surgery, Helicopter transport; Code of ED area,  National emergency department information system (NEDIS), Classification for admission through ED,  Purpose of treatment;  Patient number and type, Doctor's number,  Admission officer's doctor number; |
|  | ED sickbed information note | Information that can be obtained in the ED sickbed being a brief note in Korean. The word was made into a variable according to the frequency, and the description of each variable is as follows:  Unknown meaning missing value; Acute care unit (ACU); Acute stroke unit (ASU); Critical care unit (CCU); Neurological intensive care unit (NRICU);  General ward meaning the rest of the wards except for special departments such as ICU/ED;  Respiratory syncytial (RS); Emergency; Level; Possible; Multi-person rooms; Assignment; Preemptive; Person room; Waiting for hospitalization outside; Admission postpone; Severe; Confirm; |
| **Demographic** | Patient information | Hospital registration number, Gender, Birth, and Age |
| **Clinical** | Medical department data | Department code when making a reservation for admission (dept0), Department (dept1), Main department code in ED (dept2), Department code of admission (dept3), Department code in ED (dept4), Department code (dept5), Admission department code on the hospitalization decision form (dept6) |
|  | Hospitalization decision form | Isolation type: Unknown, Air, Protect, Droplet, Contact, and Blood |

Table S2: Description of the major variables that affect the outcome among all the variables

|  | Type | Variable Name | Description |
| --- | --- | --- | --- |
| Patient Information |  | | |
| Patient Number | Number | num | Randomly |
| Age | Number | age | 19, 20, …103, 109 |
| Gender | 2 categories | gender | Male, Female |
| Kinds of  Department code | 7 categories | dept0, dept1, dept2,  dept3, dept4, dept5,  dept6 | GI^a^, ONC^b^, OBY^c^,  CV^d^, NR^e^ … |
| Information in ED |  | | |
| ED Location Code | 17 categories | ed_loc_code | 1, 2, 3, 4(Severe level),  5, 7(Waiting for  hospitalization level),  6(Mild level), C(Cancer),  D(Delivery), R(CPR) … |
| ED NEDIS  Location Code | 7 categories | ed_nedis_code | 1.0, 2.0, … 6.0, 8.0 |
| Severe Emergency  Disease | 2 categories | severe | No(N), Yes(Y) |
| Hospitalization  Decision Check | 2 categories | adm_check | No(0), Yes(1) |
| Waiting for Admission | 2 categories | waiting | No(0), Yes(1) |
| Via ED Classification | 15 categories | via_ed_code | 11.0 (Via general ward),  28.0 (Via intensive care unit) … |
| Hospitalization  Decision Form |  | | |
| Admission  Physician Number | 577 categories | adm_dr_num | D990010, D130578,  D070498 … |
| Admission Order Date | 883 categories | adm_day | 20180614, … 20201101 |
| Diagnosis Code in  Hospital | 2811 categories | dx_code | U(Unknown),  D003017, D012602 … |
| Organ Transplant Check | 3 categories | organ | No(0), Yes(1), Unknown(U) |
| Isolation Check | 3 categories | iso | No(0), Yes(1), Unknown(U) |
| ED Admission Time |  | | |
| ED Admission Year | 3 categories | ed_y | 2018, 2019, 2020 |
| ED Admission Month | 12 categories | ed_m | 01, 02, 03, … 10, 11, 12 |
| ED Admission Date  ED Admission Time | 31 categories  1440 categories | ed_d  ed_time | 01, 02, 03, … 29, 30, 31  0000, 0001, … 2358, 2359 |
| ED Admission Hour | 24 categories | ed_hour | 00, 01, 02, … 21, 22, 23 |
| ED Admission Day | 7 categories | ed_days | Mon, Tue, Wed, … Sat, Sun |
| Admission  Reservation Time | 72 categories | adm_reserv_t | 1.0, 2.0, …  98.0 ,118.0 (hour) |

^a^_GI: gastroenterology division_

^b^_ONC: oncology-hematology division_

^c^_OBY: obstetrics and gynecology_

^d^_CV: cardiology division_

^e^_NR: neurology_

Table S3: Pre-processing results of unstructured text data. In case Korean and English are mixed in the text, the Korean meaning is written in parentheses for clarity

| **Raw data** | **Normalization** | **Tokenization** |
| --- | --- | --- |
| **Isolation type** |  | |
| 접촉주의(contact precaution) 비말주의(droplet precaution)  접촉주의+, 비말주의  공기(air), 비말(droplet), 접촉주의 | 접촉주의,  비말주의  접촉주의+, 비말주의  공기, 비말, 접촉주의 | 접촉주의  비말주의  접촉주의 비말주의  공기 비말 접촉주의 |
| **ED sickbed information note** |  | |
| GW)다인실가능(shared room available)  gw,Lv4(다인실(shared room)),rS(N)  CSICU/전(pre) PS prof  GW) VRE  GW 1O-> 타(other)FA | gw, 다인실가능  gw, lv, 다인실, rs  csicu, ps, prof  gw, vre  gw, 타fa | gw 다인실가능  gw lv 다인실 rs  csicu ps prof  gw vre  gw 타fa |

Table S4: Description of variables created by TF-IDF

|  | **Type** | **Variable Name** | **Description** |
| --- | --- | --- | --- |
| **Isolation type** | 6 categories | i0, i1, i2 i3, i4, i5 | Unknown, Air, Protect, Droplet, Contact, Blood |
| **Bed Information** | 15 categories | ACU, ASU, CCU, EM, GW, NRICU, RS, POSSIBLE, SHARED ROOM, ASSIG, FIRST, ADD BEFORE, ADD WAIT, SEVERE, CON | Acute care unit, Acute stroke unit, Critical care unit, Emergency, General ward, National emergency department information system, Respiratory syncytial, Possible, Multiple rooms, Assignable, Preemptive, Admission postpone, Waiting for hospitalization, Severe, Confirm |
